# Decoding healthcare teamwork: a typology of hospital teams

**DOI:** 10.1080/13561820.2024.2343835

**Published:** 2024-04-26

**Authors:** Natalie Sanford, Mary Lavelle, Ola Markiewicz, Gabriel Reedy, Dame Anne Marie Rafferty, Lord Ara Darzi, Janet E. Anderson

**Affiliations:** a The Florence Nightingale Faculty of Nursing, Midwifery and Palliative Care, King’s College London, London, UK; b NIHR Patient Safety and Translational Research Centre, Imperial College London, London, UK; c School of Psychology, Queen’s University Belfast, Belfast, Northern Ireland; d Faculty of Life Sciences and Medicine, King’s College London, London, UK; e Faculty of Medicine, Nursing and Health Sciences, Monash University, Melbourne, Australia

**Keywords:** Adaptive teams, team design, healthcare teamwork, interprofessional teamwork, team typology

## Abstract

The effectiveness of healthcare depends on successful teamwork. Current understanding of teamwork in healthcare is limited due to the complexity of the context, variety of team structures, and unique demands of healthcare work. This qualitative study aimed to identify different types of healthcare teams based on their structure, membership, and function. The study used an ethnographic approach to observe five teams in an English hospital. Data were analyzed using a combined inductive-deductive approach based on the Temporal Observational Analysis of Teamwork framework. A typology was developed, consisting of five team types: structural, hybrid, satellite, responsive, and coordinating. Teams were challenged to varying degrees with staffing, membership instability, equipment shortages, and other elements of the healthcare environment. Teams varied in their ability to respond to these challenges depending on their characteristics, such as their teamworking style, location, and membership. The typology developed in this study can help healthcare organizations to better understand and design effective teams for different healthcare contexts. It can also guide future research on healthcare teams and provide a framework for comparing teams across settings. To improve teamwork, healthcare organizations should consider the unique needs of different team types and design effective training programs accordingly.

## Introduction

The division of healthcare labor across multiple professionals means teamwork is an essential element of patient care (Leggat, 2007; Rosen et al., 2018; Schmutz et al., 2019). Evidence suggests that successful teamwork and optimized team composition impact outcomes such as safety (Manser, 2009), inpatient mortality (Zaranko et al., 2022), length of stay (Dutton et al., 2003), and indicators such as diagnostic accuracy, hospital acquired infection rates, and incidence of pressure ulcers (Schmutz et al., 2019). However, our current understanding of teamwork in healthcare is limited given the complexity of the context, the variety of team structures present, and the unique demands of healthcare work (Anderson et al., 2021). The study reported here is part of a larger study of adaptive hospital teams, which aimed to understand adaptive capacity and identify how teams adapt to pressures and problems related to system complexity. Early in the data collection process, it became clear that teams functioned differently in the different wards we were observing. It was therefore necessary to map these differences and understand how teams functioned before analyzing adaptive capacity. Increased understanding of the key features of differing teams is required to devise interventions that are suitable to the varied ways of working in the hospital.

Various definitions of “teams” already exist in healthcare, and all generally contend that a team consists of two or more people (Brannick et al., 1997; Freeth et al., 2008; Manser, 2009; Salas et al., 1992; Schmutz et al., 2019; Xyrichis & Ream, 2008), who share common tasks or goals (Brannick et al., 1997; Freeth et al., 2008; Manser, 2009; Salas et al., 1992; Schmutz et al., 2019; Xyrichis & Ream, 2008), who have distributed expertise (Manser, 2009; Schmutz et al., 2019), who have clearly defined roles and responsibilities (Manser, 2009; Schmutz et al., 2019), who use shared resources (Manser, 2009), and who communicate to coordinate their actions (Brannick et al., 1997; Manser, 2009; Xyrichis & Ream, 2008). Teamwork is therefore *how* people interact to accomplish work and tasks together (Schmutz et al., 2019). A number of factors are thought to improve teamwork, such as leadership, mutual performance monitoring, adaptability, the ability to support fellow team members with their work, orientation to the team, psychological safety, mutual trust, improved communication, and the use of shared mental models (Burtscher & Manser, 2012; Salas et al., 2005; Weller et al., 2014). Several barriers to achieving successful teamwork in healthcare have also been identified. These include factors such as the physical geography of the hospital and potential for teams to be dispersed, lack of interprofessional role understanding, and the potential for in-group out-group dynamics between professional groups who work closely together (Thomas, 2011; Weller et al., 2014).

Healthcare teams vary greatly on dimensions such as size, structure, location, and membership characteristics, and thus healthcare represents an opportunity to study the full range of team types found in organizations (Rosen et al., 2018). Yet, few existing studies of teamwork in healthcare adequately describe this complexity (Aloini et al., 2022). Even seemingly straightforward requirements for understanding teamwork, for instance, determining who is in the team, are nebulous in healthcare (Doekhie et al., 2017; El-Awaisi et al., 2020; Long et al., 2006; Sorensen et al., 2008). Equally, the type of interprofessional work required by the team can differ depending on the context and may involve teamworking, networking, collaborating, or coordinating (Reeves et al., 2018; Xyrichis & Ream, 2008; Xyrichis et al., 2018). Existing research on healthcare teams generally focuses on one team at a time and typically includes teams who are self-contained and easier to define, for instance cardiac arrest or surgical teams. Less is known about the diversity and complexity of teamwork on wards, and even between ward teams, there is structural variation (Kannampallil et al., 2011; Lingard et al., 2017). Until we understand how different teams function and what their needs are, our understanding of the larger system of teams throughout the hospital will remain incomplete, interventions to improve teamworking will continue to fall short, and both patient quality and safety outcomes and organizational metrics will be impacted.

## Existing team typologies

While others have previously recognized that there are many types of teams in healthcare (Manser, 2009; Mitchell et al., 2012; Rosen et al., 2018), only a few typologies for classifying teams in healthcare currently exist. Furthermore, these existing typologies do not have the specificity required to capture the complexity of or to meaningfully differentiate between healthcare teams. For example, one typology separates team types by their collective working processes (Klarare et al., 2019; Thylefors et al., 2005). Yet, with only three types of teams (multiprofessional, interprofessional, and transprofessional), this typology does not have the granularity to distinguish between healthcare teams that differ based on other features, such as team location or membership stability. A second typology identifies teams by whether the role and personnel are stable or variable (Andreatta, 2010). However, these select membership features also lack the specificity to differentiate healthcare teams, as many in-patient hospital teams would fit into the stable role, variable personnel category. Finally, the World Health Organization identify five team types, differentiated by their type of work: core teams, coordinating teams, contingency teams, ancillary services, and support services and administration (Babiker et al., 2014; World Health Organization, 2012). While this typology differentiates teams by categorizing the type of work they do, it does not capture the complexity of and variation between different types of “core teams,” who all deliver clinical care but who may not all be structured the same way. Furthermore, the typology is not empirically derived.

Teamwork is a fundamental component of healthcare provision (Leggat, 2007; Manser, 2009). Yet, to date, an understanding of how different types of teams differ, interact with each other, and contribute to the overall effectiveness of healthcare work in the hospital is currently missing. The first step to understanding teamworking across the hospital is to establish what types of teams exist to enable empirically studying their features, differences, and how they interact with each other. What is needed is a typology that accounts for multiple elements of team design. This paper presents a new typology derived from empirical data that incorporates multiple distinguishing features, including structural, membership, and functional elements in differentiating healthcare teams.

## Aims

The aims of this study were to:

1. Identify different types of healthcare teams based on their structure, membership, and function; and
2. Identify whether and how teamwork behaviors differed between team types.

## Methods

### Design

As reported in Sanford et al (2021, 2022a, 2022b), a research team of clinicians and non-clinicians from different backgrounds, including social science, psychology, human factors, nursing, medicine, patient safety, and education, conducted non-participant ethnographic observations between October 2018 and March 2019.

### Data collection

Authors ML and OM undertook 88.5 hours of non-participant ethnographic observations in a large hospital in central London. The hospital in which the study took place is a major trauma center and teaching hospital. Easily accessed by public transportation and serving a large, diverse population of Londoners, the hospital offers a wide range of specialized services, including cancer care, cardiology, and a 24-hour emergency department. The hospital contains over 700 beds and treats over one million patients annually. Purposive sampling was used to select five diverse ward areas, representing a variety of in-patient hospital environments. The wards included in the study were: two surgical wards, an older adult ward, a critical care unit, and the Acute Assessment Unit (AAU), a unit to expedite patient flow out of the Emergency Department (see Table 1).

**Table 1.** Wards observed, duration of observation per ward, and description of ward areas.

Clinical Area	Total Duration of Observation (hours)	Description of Area
Surgical wards	35	Multi-bay wards with patients who are managed by surgical teams
Older Adult	23	Multi-bay wards with elderly patients who are managed by a team specializing in frailty and geriatrics
Critical Care	7.5	Multi-bay ward with critically ill patients. Patients are managed by a specialized critical care team
AAU	23	Unit consisting of multiple multi-bay wards with patients cared for by a medical team. Patients have a maximum stay of 48-hours before being either discharged from the hospital or transferred to another ward

Events were observed as they occurred naturally, and observations were not confined to the ward area. These observations included activities such as ward rounds, medication rounds, and general ward activities, as well as coordinating events such as handovers, board rounds, multi-disciplinary team (MDT) meetings, and bed availability meetings. In-depth ethnographic field notes were transcribed by hand with identifying information removed. These were transcribed electronically immediately after observation by the observer and were uploaded into NVivo 12 for storage, organization, and analysis on a password-protected computer (Sanford et al., 2021, 2022a, 2022b).

### Data analysis

Data analysis involved a combined deductive-inductive approach to thematic analysis, which was completed in three iterative phases between July 2020 and March 2023. In phase one, after initial data immersion, deductive codes based on the Temporal Observational Analysis of Teamwork (TOAsT) framework were applied (Lavelle et al., 2020). The TOAsT framework is a reliable, empirically derived behavioral coding framework for observable interprofessional teamwork behaviors. The framework consists of five teamwork domains: monitoring team performance; team attitudes; leading the team; developing a shared mental model; and requesting and providing assistance. These domains are further specified with corresponding behavioral functions and observable behaviors. For instance, for the domain of leading the team, a behavioral function is delegating, with observable behaviors of instruction and workload redistribution. All elements on all three levels (domains, behavioral functions, and observable behaviors) were added as hierarchical codes in NVivo. The transcripts were coded by author NS to identify segments of text that demonstrated instances of the TOAsT observable behaviors. The codes were then aggregated under their corresponding behavioral functions and behavioral domain parent codes. The deductive codes were discussed during regular team meetings to ensure conceptual agreement was present throughout the analysis process, with frequent reviewing of the data and sense-checking of the coding. The team’s combined expertise in healthcare (NS, OM, AMR) and the TOAsT framework (ML, GR, JA) was essential to developing a shared understanding of healthcare teamwork behaviors.

Next, in phase two, the research team inductively analyzed the transcripts, considering team structure, membership, design, function, and location to devise a typology of different team types. Although only five clinical areas were studied, those teams often interacted with other teams throughout the course of their clinical work, for instance, the bed management team, the rapid response team, and specialist consulting services like cardiology, endocrinology, and infection control teams. These teams were also considered during typology development. Ultimately, five team types were derived from the data: structural, hybrid, satellite, responsive, and coordinating.

Finally, in phase three, these five team types were embedded into NVivo as “Cases” by NS to enable the comparison of TOAsT teamworking behaviors across team types via a paired, matrix coding query. The matrix coding query function is a search function that enables the identification of text coded as each TOAsT domain, function, and behavior within each Case category. This allowed us to collate summaries of each teamworking behavior for each team type. These excerpts were reviewed in detail by NS and JA, who synthesized these into narratives. The synthesized data were then reviewed by the entire team during team meetings for further discussion and interpretation.

### Ethical considerations

As previously described in Sanford et al (2021, 2022a, 2022b) the study received ethical approval (REC REF:18/WA/0218) and formal approval from Trust leads. The lead doctor(s) (e.g., consultant) and lead nurse(s) (e.g., ward matron) in each participating ward area received information about the study through verbal presentations and written study information sheets. For a ward to be included in the study, written informed consent was required from all clinical leads on that ward. Consent was also obtained from staff to allow researchers to observe their routine work. A total of 36 healthcare staff members provided written informed consent to participate, allowing for data collection of routine work across five ward areas.

## Results

In the following sections, we first present an overview of the main features of each team type, followed by an analysis of teamwork behaviors in each type we observed.

### Typology of teams

**Figure 1.**
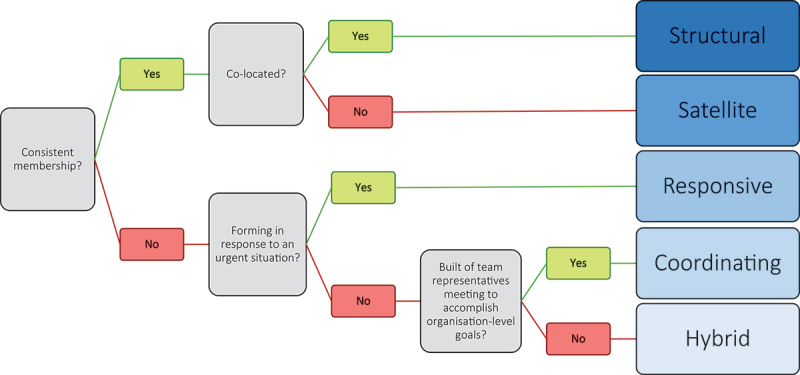
Team typology decision tree.

Teams differed in their structure, membership, and function. Structurally, we observed that teamwork could have either a sustained or episodic style depending on the team and that teamwork could take place in either a fixed or mobile location. These elements capture the temporospatial possibilities for teamworking. Team membership also varied between teams; team members could be stable (always belonging to that team) or unstable (joining and re-joining the team when sharing tasks). The function of the team- the tasks they do, goals they have, and whether the team are co-located together- also varied. Based on these factors, five different types of healthcare teams were identified: structural, hybrid, satellite, responsive, and coordinating. Table 2 provides an overview and description of each team type and Figure 1 can be used to help identify each team type in practice based on their distinguishing structure, membership, and function features.

**Table 2.** Description of team types.

Team Type	Teamworking Style	Location	Membership	Description of Team and Tasks	Team(s) Observed in this Study
Structural	Sustained	Fixed	Stable membership	Ward-based teams who provide routine inpatient care with all members of the team consistently co-located together on the ward	Geriatric Ward; Critical Care Ward
Hybrid	Sustained	Fixed	Some stable members and some unstable members	Ward-based teams who provide routine inpatient care with only some members of the team consistently co-located together on the ward	Acute Assessment Unit
Satellite	Sustained or episodic	Mobile	Stable membership	Mobile teams who provide consulting clinical care (such as monitoring, assessing, and care planning) for patients on multiple wards, whilst coordinating with the primary ward team	Surgical team
Responsive	Episodic	Mobile	Unstable membership	Mobile clinical teams formed episodically to respond to deteriorating patient condition, such as cardiac arrest	Not observed
Coordinating	Sustained	Fixed	Some stable members and some unstable members	Office-based teams who manage organizational goals, challenges, and priorities. This team can also include representatives from multiple teams across the hospital to exchange information and plan	Bed management team; patient flow team

The following sections present the results for the teams we observed in practice and their teamworking behaviors.

### Structural team

The structural teams were based on a ward for the care of the elderly and a critical care unit. The multi-disciplinary team members we observed included doctors, nurses, healthcare assistants, dieticians, and physiotherapists. These professionals provided specialist services in all aspects of elderly care including falls, frailty, cognitive problems, and general rehabilitation. In critical care, services included assessing and treating high acuity patients who require close monitoring. In both wards, teamwork was sustained and took place in a fixed location, members were co-located, and membership was stable.

The structural teams were characterized by high levels of trust between team members. Team members were observed to discuss uncertainties and gaps in knowledge and to ask for feedback from others about their decisions. For example, following a difficult discharge that involved disagreement and negotiation with a care facility, the discharge nurse on the geriatric ward was observed to say that the whole process had left her feeling unsure of her assessments. As such, she asked the other members of the team for feedback on her work. A senior colleague said he would collaboratively review her assessments with her to see if they had shared agreement. Team members also offered each other support in difficult situations, often by redistributing or sharing tasks. In one such situation, a nurse and a junior doctor worked together on taking patient observations, with one taking the vital signs whilst the other confirmed which observations were required and updating the information in the medical record when it was collected. There was a high level of information sharing and discussion about patients and the team usually agreed on the best care plan for each patient, keeping each other informed about changes and roadblocks to plan execution. There were many opportunities for learning and reflection during team meetings and other interactions, with senior clinicians taking time to teach and reflect on the progress of care. Ward rounds were interprofessional and bedside nurses and other allied health professionals were often proactively included, asked for information, consulted, and updated on care plans.

### Hybrid team

The hybrid team was based on an acute assessment unit (AAU). The AAU was a short-stay unit to expedite patients out of the Emergency Department (ED). The ward had a multi-disciplinary team of doctors, nurses, healthcare assistants, and other allied health professionals. Although the clinical duties on this ward were like those of the care of the elderly structural team, the ward was staffed by several teams of doctors. The doctors had unstable team membership and were not consistently co-located with the other team members on the ward. Therefore, there was high reliance on the ward manager, who coordinated patient care and facilitated information exchange with the doctor teams. AAU work was characterized by constant interruptions and requests for information. This mostly affected senior nurses, who were familiar with the unit and the patients, and who became an information source for unstable team members who were not familiar with or consistently located on the ward. The strategy for optimizing efficiency was that it was quicker to ask the charge nurse or nurse manager for information than to grapple with unfamiliar and unwieldy IT systems. Medical staff seemed to prefer to approach a specific nurse they were familiar with for information when necessary and were not observed to include bedside nurses in their ward rounds nor update bedside nurses after ward rounds.

The high patient turnover on AAU required continuous coordination of transfers, discharges, and admissions. Frequent patient movement led to increased pressure for the team and additional challenges. For example, patients were often brought to the ward from the ED as soon as they were assigned a bed in the AAU, generally without a proper handover. This was done to free up space in the ED as soon as possible, and to avoid breaching the 12-hour ED stay target. However, in these cases, the patients were not known to the AAU team and information was often missing or incomplete, creating more demands and requiring the team to do investigatory work to learn about the patient. Furthermore, to expedite transfers out of the ward and discharges, stable AAU hybrid team members (usually the nurses) had to coordinate patient care with other wards, departments, and services, liaising with a wide range of other professionals across the hospital to move patients along their care pathway more quickly to free-up space on the AAU. This could involve calling radiology to expedite a discharge-dependent scan, team members transferring patients or cleaning bays instead of waiting for porters or domestic staff, and proactively phoning the unstable team members (doctors) to request completion of discharge letters, ward round documentation, and orders. Finally, many patients were admitted to the AAU early in their hospital stay for assessment before being assigned to an in-patient medical ward. Because of this, nurses on the AAU often knew where patients were located throughout the hospital, even after they were no longer in their care. Other teams were aware of the AUU team’s knowledge, and frequently called upon the nurses to ask where their patients had been transferred to. Senior nurses were observed answering and directing large numbers of calls from across the hospital about patients who had been admitted to and subsequently transferred out of the AAU. Calling the AAU for information was faster for the caller than locating patients electronically or using the formal switchboard, but created additional, unnecessary work for the team. Altogether, the AAU team appeared to work at high intensity and often worked through breaks and past the end of their shift. The overall impression was that hybrid teams were under resourced for the work required.

### Satellite team

The satellite team observed in this study was a surgical team who had patients located on many wards throughout the hospital and traveled around the hospital completing ward rounds. The team was sometimes uni-disciplinary, involving multiple doctors of various seniority levels, and other times was multi-disciplinary, involving doctors as well as an advanced surgical nurse practitioner. The team had a large, geographically diverse ward round to complete, which limited time to complete the tasks required for each patient, such as ordering tests, prescribing medications, documenting details of the round, and arranging discharge documentation. The team was often observed dividing up tasks to make the ward round more efficient. However, division of tasks during the ward round was sometimes ambiguous generating the risk that some tasks would not get completed. For example, when a decision to discharge a patient was made, one team member would begin the discharge summary during the ward round, but it was not always clear who would complete it, sign it off, and communicate the plan to the bedside nurse. If the team split up to complete tasks in different areas of the hospital using a “divide and conquer” approach on days when the patient list was long, team members lost access to information about some patients and the decisions made about their care. Team members used a messaging application on their personal mobiles to communicate when they were in different parts of the hospital, resulting in frequent notifications, and the need to monitor multiple communication platforms (the electronic patient record, bleepers, the messaging application, and written notes) simultaneously.

Furthermore, the team faced many challenges with equipment. Because they traveled around the hospital, they often experienced equipment shortages such as lack of access to ward computers, which resulted in them making paper notes which needed to be transcribed later. Lack of computer access also led to decisions being made without up-to-date information, such as the latest test results and scans. In some wards there appeared to be difficulty negotiating access to IT systems and equipment, with ward staff appearing to protect their own resources, for example, putting a written notice on a computer stating, “for X person’s use only.” Interruptions were common. Lack of privacy and space to discuss patients on each ward also affected the team’s ability to discuss patient needs and agree a way forward. Many conversations about care decisions occurred in the corridor and stairwells moving between wards. In some cases, the team was observed being told to be quiet to avoid disrupting patients, which obstructed their conversations. Ward staff were rarely engaged by the team to join ward rounds or to exchange information after the round was complete. Some nurses and allied health professionals sought out the team to ask questions or raise concerns, but interactions were largely opportunistic rather than a structured part of the process. Conversations with nurses were more common in specialty wards where the satellite team frequently had patients and thus were more familiar with the local team.

### Responsive team

As mentioned previously, the original aim of the larger study did not involve mapping differences between team types. This analysis developed after data collection had started, so our recruited teams do not capture all the types in the typology that this study yielded. As such, we did not directly observe the responsive team. Still, due to the episodic, mobile nature of their work, we briefly encountered the responsive team on several occasions during our observations, enabling us to incorporate this team type into the typology. The responsive team we encountered was a cardiac arrest team, which appeared to be made up of senior clinicians from around the hospital who carried a bleeper to which cardiac arrest calls would be put out in the event of a cardiac arrest. Each clinician had a primary team they belonged to, and the team members were dispersed throughout the hospital working in different areas before responding to a call. In the event of a cardiac arrest, the bleeper would sound, and the team members would unite episodically to treat the deteriorating patient. We observed this first hand during our observations of the hybrid team, when a hybrid-team doctor received a cardiac arrest call to their bleeper and was pulled away from ward rounds to attend to an arrest.

### Coordinating team

The final team type we observed was the coordinating team. We observed two such teams during our shadowing of clinical leads from other areas: a patient flow team and a bed management team. The coordinating teams were made up of some stable and some unstable members with a fixed, meeting room/office location. Stable members included bed managers, site operation managers, and hospital executives. Unstable members included ward representatives (typically ward managers or nurses in charge) and other professionals (estates and facilities manager, clinicals leads, etc) from around the hospital. Our observations took place during pre-organized encounters, such as bed, quality improvement, and flow meetings. We did not observe the coordinating teams’ teamwork outside of these meetings during this study. During the meetings, representatives from different areas attended to problem solve, update information, and co-ordinate responses. Most meetings were focused on:

- Sharing the latest information from different wards, for example, staffing levels and potential discharges
- Verifying centrally held bed availability data for accuracy and updating with the latest intelligence from the wards
- Ensuring the right people were in attendance, for example, porters and cleaners were often absent despite being seen as important for solving problems
- Connecting people from different areas who could provide help for a specific problem
- Agreeing on the problem to be solved and what actions were appropriate

Because members of this team came from disparate ward areas and were reporting to the stable team members, the full team had limited opportunity to build a shared mental model before meetings. Much of their initial discussions were focused on building this shared understanding, although it wasn’t always clear whether agreement had been reached in the discussion before there was pressure to move on. The team grappled with basic goal conflicts in the hospital system that they did not have the power to change. For example, a common focus of discussions was the difficulty of coordinating patient flow with the cleaning and portering staff who were employed by a third-party facilities management company. Similarly, goal conflicts were observed to occur for team members who defended their ward’s performance and at times argued for further resources. Balancing the needs of individual wards with patient flow and bed management for the whole hospital was challenging. The team interactions had a friendly open communication style, but the meetings were more formal than those of other teams and there were few humorous or lighthearted exchanges observed. There was a sense that the organization was operating at a high level of pressure all the time so there was little room to maneuver.

### Differences between team types

There were several key differences in teamwork behaviors observed between the different team types. First, of the ward-based teams (structural, hybrid, and satellite) the structural team was the most interprofessionally collaborative. The team had clear and distributed leadership that involved rationale dissemination, delegating, planning, and information gathering. The team exhibited behaviors associated with shared mental model development through frequent information sharing and speaking up. Likewise, the satellite team exhibited similar leadership and shared mental model development behaviors within their team. The hybrid team seemed to lack clear leadership and instead involved senior, stable team members (usually the nurse in charge) chasing information from unstable team members and responding to requests for information from unstable team members. Outside of these spontaneous interactions, there was limited interaction between stable and unstable team members, which inhibited shared mental model development. The hybrid team seemed to function as two, disjointed teams, rather than one team working together.

Requesting and providing assistance was characteristic of both the hybrid and satellite teams. However, unstable hybrid team members were not observed to help stable hybrid team members or vice versa, and this assistance largely took place between co-located team members. Other team behaviors, such as monitoring team performance and team attitude behaviors, were more difficult to extrapolate from the data; these behaviors may occur less frequently or be more difficult to observe.

The coordinating team rarely exhibited any of the teamworking behaviors apart from information gathering and information sharing. The formal nature of the meetings we observed may have contributed to this being the case. The implicit purpose of the observed meeting was to gather and share information for subsequent planning and delegation but planning and delegation activities were not directly observed.

## Discussion

In this exploratory study of healthcare teams, we identified five different types of teams that vary in structure, membership, and function: structural, hybrid, satellite, responsive, and coordinating. Structural teams, in which people who worked together regularly in a shared location, appeared to have more ability to implicitly coordinate their activities, communicate effectively, and manage their workload through division of labor and intra-team support. We hypothesize that the highly effective teamwork they exhibited relied on their stable membership, familiarity with each other, well-designed and practiced coordinating activities, and the fact that they were co-located. Other teams experienced challenges that made effective teamwork difficult. The hybrid team lacked integration between the stable members of the team and unstable members, resulting in a high workload for the stable members to compensate by trying to bridge the communication gap. The satellite team experienced challenges in coordinating their activities in diverse locations with no ownership of space or equipment. The coordinating team had to balance the competing goals of optimizing organizational performance and optimizing unit or ward performance. This inhibited open communication, and the focus of meetings was mostly on information gathering and exchange rather than co-ordinating activities. Although the structural team also experienced challenges, they appeared to have less effect on teamwork than in the other teams.

Other elements of the healthcare work system also complicate teamwork. For example, unlike teams in most other industries, healthcare teams are unique in that one individual may be part of multiple teams simultaneously, each with a different structure, location, and culture (Manser, 2009; Mitchell et al., 2012; Rosen et al., 2018). High staff turnover is also a feature of healthcare, especially since the pandemic (Dinh et al., 2021; Rangachari & Woods, 2020). Although previously teams could integrate new members gradually without changing the core membership significantly, high turnover means many members do not know each other. Previous research has suggested that membership stability is crucial to teamworking (O’Leary, 2016), so instability in teams that previously maintained relatively stable membership is likely to have implications for both outcomes and team culture. Indeed, a recent study on nurse staffing and inpatient mortality found that agency nurses are not a like-for-like substitute for local staff (Zaranko et al., 2022).

Suggested strategies for overcoming teamworking barriers in healthcare often include training teams together, incorporating teamwork into educational curriculums, increasing team democracy and inclusivity, and developing structured communication techniques (Weller et al., 2014). However, some of these interventions may not be possible or suitable for all healthcare teams. For instance, for teams who are not co-located, opportunities for training may be limited. Some teams are formed ad hoc in response to situational demands, and thus may have familiarity with one another’s roles but may never have met prior to the teamworking interaction, such as a cardiac arrest team (responsive). Some teams, such as the hybrid team, may not operate as an integrated team so training that assumes a well-defined team exists may not be suitable. The satellite team may need support in integrating into ward teams for the time they are on the ward. Understanding these differences between teams, and raising awareness amongst staff of how teams, and therefore teamwork, differ is crucial for designing effective training. This understanding may help further explain the results of a previous review on interprofessional collaboration, which highlighted inconsistent findings in team impact on patient, staff, and organizational factors (Pomare et al., 2020).

The finding that stable membership and co-location of team members facilitates teamwork should not be surprising given that coordination itself requires work and this work is increased in the absence of the time and resources to build, maintain, and repair common ground (Klein et al., 2005). Effective coordination relies on the ability of all team members to reliably predict the actions and requirements of others; to develop, maintain, and repair common ground; and to direct others and take direction from them. Common ground is the shared knowledge, beliefs, and assumptions that allow teams to coordinate their activities and it requires time to establish (Klein et al., 2005). The results of this study suggest that these activities are all easier for teams who are co-located and familiar with each other; otherwise, the time, effort, and resources required for coordination increase. This is especially the case if common ground is lost. In hybrid teams, common ground may be especially difficult to build, maintain, and repair without explicit mechanisms that support it. These mechanisms could include clear procedures; induction and orientation; huddles to update incoming team members; and better support in the workplace for unfamiliarity with the environment. Given that staff shortages, rapidly forming teams, redeployment, and temporary and contract positions are increasingly common, our findings suggest that healthcare organizations could better support teamwork by providing sufficient resources to maintain coordination according to the demands experienced by different teams.

### Practical implications

We identified many challenges for teams that could be reduced by considering the unique needs of different team types. The satellite team was frequently challenged by lack of equipment, which could be resolved by providing equipment for their use. The hybrid team was challenged by the unstable team members being unfamiliar with many aspects of the ward environment such as operating devices and finding equipment. Good organization and design of the workspace to support those unfamiliar with the environment could mitigate some of these problems, as well as robust induction and communication processes. The typology presented in this paper provides a framework for thinking about team differences and how efforts to improve teamwork should be tailored to their needs.

We recommend that the typology we have developed be used as a sensitizing framework for identifying team differences and requirements for improvement. It is not intended to be static and prescriptive, because how a team is defined depends on the perspective and aims of the observer. In this study, we identified teams by shadowing individual clinicians and observing how and who they worked with. The fluid and dynamic network of teams that exist in healthcare mean that teams could be classified differently, depending on the focus of the analyst. For example, the satellite team was observed as it traveled around the hospital, but at times when the satellite team was interacting with a ward team, this larger team could also be viewed as a hybrid team. In this study we were interested in how the satellite team coordinated its own activities to complete ward rounds and so defined it as a satellite team. The complexity of healthcare teamwork means that studying teams in situ is challenging and varies depending on the context (Pomare et al., 2020). Defining the team, understanding interactions between members, and interactions between teams, means we need theoretical perspectives that attend to the team context and how it shapes work. This typology, which has emerged from exploratory research embedded in one acute care hospital, provides a basis for differentiating teams and tailoring training and organizational support to the team’s needs. Further exploration and development of the typology is necessary, and its utility has already been demonstrated in a study to explore the role of leadership in hospital teams in Norway (Fagerdal et al., 2022) and to examine resilience and teamwork in a multinational study (Anderson et al., 2020). In future research, there is opportunity for this typology to be paired with existing tools that explore the work and teamwork behaviors of interprofessional teams, such as the InterProfessional Activity Classification Tool (Xyrichis et al., 2018) and the Temporal Observational Analysis of Teams framework (Lavelle et al., 2020), among others. We argue that typifying teams prior to quantitatively or qualitatively analyzing their work could enable deeper insight into teams’ strengths, support needs, and unique functionality, as well as enabling robust inter-team comparison.

### Limitations

As previously disclosed, the original purpose of this project was not to identify types of healthcare teams. As such, we did not directly observe every team type represented in the typology. It is possible that there are additional types of teams not included in this typology. For example, the increased use of telehealth consulting and remote collaboration suggests there may be a need to include remote or virtual teams. Future research should involve further analysis of all team types and should especially further explore the features of responsive and coordinating teams. Data were collected in one hospital only, but it is unlikely that this hospital differed significantly from others. Given the qualitative, ethnographic methods used, we did not aim for generalizability of the typology, but offer it as an interpretive framework to think about and understand teams. Further research in a range of different types of organizations, such as community and mental health teams, is necessary to capture the full range of healthcare teams. Finally, the data were collected prior to the COVID-19 pandemic, which may have changed team types and behaviors. Further study using the typology is needed and encouraged so that the complexities of healthcare teams and change over time can be decoded.

## Conclusion

This paper presents a new typology of healthcare teams based on their structure, membership, and function. It is the first typology with the granularity to differentiate between different types of clinical teams based on these features. The typology provides a lens for observing and understanding teams and teamwork, and a language with which to build a shared understanding of teamwork, which is the first step toward teamwork improvement.
